# Progressive muscle relaxation increases slow‐wave sleep during a daytime nap

**DOI:** 10.1111/jsr.13574

**Published:** 2022-03-30

**Authors:** Katharine C. Simon, Elizabeth A. McDevitt, Rocco Ragano, Sara C. Mednick

**Affiliations:** ^1^ Department of Cognitive Science University of California Irvine California USA; ^2^ Department of Psychology Princeton Neuroscience Institute Princeton University Princeton New Jersey USA; ^3^ Self‐employed

**Keywords:** electroencephalography (EEG) lateralisation, napping, progressive muscle relaxation, restorative processes, sleep architecture, slow‐wave sleep

## Abstract

Sleep is critical for health, cognition, and restorative processes, and yet, many experience chronic sleep restriction. Sleep interventions have been designed to enhance overnight sleep quality and physiology. Components of these interventions, like relaxation‐based progressive muscle relaxation (PMR), have been studied in isolation and have shown direct effects on sleep architecture, including increasing time in restorative, slow‐wave sleep (SWS). These relaxation methods have been understudied in naps, which are effective fatigue countermeasures that reduce deleterious effects of chronic sleep restriction. We hypothesised that PMR should boost SWS in a nap, as compared to an active control. We used a between‐subject design in which healthy young adults underwent PMR training or listened to Mozart music (control) prior to a 90‐min nap opportunity. We assessed changes in the amount and lateralisation of SWS, as evidence suggests left hemispheric lateralisation may be a proxy for recuperative sleep needs, and changes to state‐dependent anxiety and fatigue before and after the nap to assess intervention success. We found PMR participants spent ~10 min more in SWS, equivalent to 125% more time, than the control group, and concomitantly, significantly less time in rapid eye movement sleep. PMR participants also had greater right lateralised slow‐wave activity and delta activity compared to the control suggesting a more well‐rested brain profile during sleep. Further, pre‐sleep anxiety levels predicted nap architecture in the intervention group, suggesting benefits may be impacted by anxiety. The feasibility and accessibility of PMR prior to a nap make this an interesting research avenue to pursue with strong translational application.

## 1 INTRODUCTION

While sleep may have many functions, one fundamental purpose appears to be restoration (Benington & Heller, 1995; Borbély, 1982). The accumulating weight of waking activities requires a sufficient period of time for our brain and body to be restored to baseline functioning (Borbély, 1982; Tononi & Cirelli, 2014). Despite this necessity, a large portion of the population experiences chronic sleep loss or frequent sleep disruption due to a range of underlying reasons including shift work, low priority to sleep, parenting, sleep disorders, or psychological factors (Åkerstedt, 2003; Banks & Dinges, 2007; Costa, 1996). Consequences of poor sleep are far‐reaching and can include worse immune functioning, greater cardiovascular disease, cognitive deficits, attention lapses, and emotional dysregulation (Banks & Dinges, 2007; Krause et al., 2017). Napping has been shown to benefit chronic sleep loss, daytime fatigue, and is an effective tool for managing disrupted sleep (Bonnefond et al., 2001; Bonnet, 1991; Costa, 1996; Härmä et al., 1989; Macchi et al., 2002; Vgontzas et al., 2007). Moreover, napping shows a wide range of immediate daytime benefits such as enhanced mood, attention, perceptual learning, and memory (Chen et al., 2020; McDevitt et al., 2018; Mednick et al., 2002, 2003, 2008; Milner & Cote, 2009). Although naps are not recommended for those with sleep disorders undergoing insomnia treatment, a large portion of the population naps for a variety of reasons, spanning restoration from fatigue, prophylactic for upcoming sleep loss, enjoyment, termed appetitive, or emotional reasons, typically for those with higher mental health symptoms (Dinges, 1992; Duggan et al., 2018). Despite the benefits, though, napping appears to be underutilised for restorative purposes and at times difficult to attain by the current population (Duggan et al., 2018; Faraut et al., 2017). A number of relaxation‐based interventions have been devised to facilitate night‐time sleep; however, they have been infrequently investigated in the context of daytime napping (Cordi et al., 2019; Debellemaniere et al., 2018).

Accessibility to sleep interventions administered by experts can be limited, but relaxation methods, common components of psychological and behavioural sleep interventions, are easily available and can be equally efficacious at home (Morin et al., 2006). The spectrum of stress‐reducing, relaxation techniques can be non‐specific or target physiology or cognition. Research supports their efficacy in decreasing physiological arousal, stress, reducing self‐reported anxiety, and improving self‐reported sleep quality (Robb, 2000). Physiological relaxation techniques, such as progressive muscle relaxation (PMR), have been shown to alter sleep architecture prior to overnight sleep. In PMR, individuals are trained to enhance awareness of their bodies’ muscle tension and systematically tense then release their large muscle groups (Conrad & Roth, 2007; McCallie et al., 2006). This muscle contraction‐release technique alleviates overall muscle tension and effectively increases global physiological relaxation, potentially related to PMR’s enhancement of parasympathetic activity (Dolbier & Rush, 2012). Physiological benefits of PMR include improved heart rate, cortisol and blood pressure, and pain while psychological benefits include stress and anxiety alleviation (Conrad & Roth, 2007; Krajewski et al., 2011; McCallie et al., 2006) When used before night‐time sleep in both typical and clinical populations, PMR consistently improves self‐reported rates of relaxation, reduces pre‐sleep anxiety, decreases sleep onset latency (SOL), and alters sleep architecture, specifically increasing the total time spent in slow‐wave sleep (SWS) (Sun et al., 2013).

Alternatively, non‐specific relaxation methods, such as listening to music, consistently results in enhanced self‐reported rates of relaxation, reduced anxiety, and improved self‐reported sleep quality, including decreased SOL, enhanced self‐reported sleep quality, and improved sleep efficiency (Feng et al., 2018; Jespersen et al., 2015; De Niet et al., 2009; Robb, 2000). Despite subjective benefits, few studies have shown objective improvements in sleep architecture using these non‐specific relaxation methods. In one recent study by Cordi et al., listening to music prior to a nap reduced the time spent in non‐rapid eye movement (NREM) Stage 1 (N1) and increased the time spent in SWS; however, this was only for participants who were rated highly on a suggestibility index (Cordi et al., 2019). In contrast, a study by Chang et al. found that repeated soothing music before night‐time sleep increased rapid eye movement (REM) sleep, decreased N2, and had no effect on SWS (Chang et al., 2012).

One way to evaluate the success of interventions to support restorative sleep is to assess sleeping brain rhythms. Considerable evidence has shown that the time spent in and the power of SWS are markers of homeostatic sleep pressure, akin to restoration needs (Borbély, 1982; Riedner et al., 2007). Both the time spent in SWS and low electroencephalography (EEG) frequency power directly correlate with the preceding amount of time awake (Achermann et al., 1993; Borbély, 1982; Borbély et al., 1981; Cajochen et al., 1999; Finelli et al., 2000). Consistent evidence has also shown that in SWS, left‐lateralised delta power (1–4 Hz) may also be a proxy for recuperative sleep needs (Achermann et al., 2001; Ferrara et al., 2002; Kattler et al., 1994). Achermann et al. found increased left lateralised frontocentral delta activity after sleep deprivation (Achermann et al., 2001). Similarly, Ferrara et al. showed that after 2 nights of sleep deprivation, the subsequent sleep episode showed a greater left‐lateralised rebound effect (Ferrara et al., 2002). Furthermore, recuperative requirements appear to be greater for the left compared to right hemisphere as shown by Kattler et al. (Kattler et al., 1994). Their study demonstrated that right hand stimulation before sleep would result in increased left‐lateralised delta activity during SWS, but the opposite, increased right‐lateralised activity when stimulating the left hand, was not found. Altogether, these findings suggest that experience‐dependent exhaustion of neural circuits during waking activity may be reflected by greater left lateralised low frequency activity during SWS and evaluating EEG lateralisation may provide insight into sleep’s restoration functions.

A second way to evaluate the success of a sleep‐based intervention is through assessment of subjective mood rating changes. Sleep is theorised to play a key role in supporting psychological health by modulating emotional experiences, such that emotional reactivity is reduced following sleep (van der Helm et al., 2011; Walker & van der Helm, 2009). Anxiety is a hallmark of sleep disorders, and sleep disturbances are known to both impact and be impacted by anxiety symptoms (Cox & Olatunji, 2016). A recent meta‐analysis discovered that overnight sleep deprivation appears to be causally related to anxiety symptoms (Pires et al., 2016). In line with this, Horváth et al. (2016) investigated how state and trait levels of anxiety were related to changes in sleep architecture using the State‐Trait Anxiety Inventory (STAI). They found that heightened levels of both state and trait anxiety were correlated with an increase in Stage 2 and decrease in SWS, and higher trait levels of anxiety were associated with reduced total REM sleep (Horváth et al., 2016). In line with these sleep findings, PMR has also been associated with reductions in anxiety in young adults, pregnancy, patients with breast cancer, and patients with coronavirus disease 2019 (COVID‐19) (Liu et al., 2020; Gok Metin et al., 2019; Powell, 2004; Rajeswari & SanjeevaReddy, 2019; Wilczyńska et al., 2019). As such, the prior literature suggests an anxiolytic benefit should be heightened after a combined nap and PMR intervention.

Given the immediate cognitive, psychological, and physiological restorative benefits of a daytime nap, we hypothesised that a PMR intervention before a nap would increase the total time spent in SWS compared to an active non‐specific relaxation control group that listened to classical music. We also hypothesised that the PMR would reduce the required recuperative needs of sleep, as evidenced by reduced left‐lateralisation low‐frequency EEG compared to a greater left‐lateralised activity in the control group. We used a between‐subject design to test our hypothesis with half our participants following an audio‐taped PMR training before a nap while the other half listened to an equal‐length audio tape of a Mozart medley of classical music. We evaluated the differences in sleep variables between the groups, including total sleep time (TST), SOL, wake after sleep onset (WASO), the total time spent in each sleep stage, power spectra, and laterality of low‐frequency EEG. We also evaluated intervention success by measuring state‐dependent anxiety and fatigue levels both before and after the nap. If successful, PMR could be adopted to enhance the benefits of daytime napping.

## 2 METHODS

### 2.1 Participants

A total of 50 (24 female) college‐aged students (mean [SD] age 20.17 [1.86] years) participated in our sleep study. Prior to participation, we administered oral and written informed consent. Our study and procedures were approved by the Institutional Review Board of the University of California, San Diego. Participants received course credits or monetary compensation for their time. Participants were healthy, non‐smoking, young adults with no history of neurological disorder, psychological disorder, chronic illness, substance use history, or sleep disturbance history. All participants were required to have consistent sleep/wake cycles and have typical sleep the night before participation.

We conducted a power analysis in g*power using one‐way analysis of variance (ANOVA). We found that with a total sample size of 34 participants, our study had 80% power to detect a medium between‐subject effect. Given that some participants had difficulty napping, we recruited participants until we reached our required sample size. All participants were randomly assigned by alternating the intervention (PMR, *n* = 24) or control (music, *n* = 26) conditions as participants were recruited. A total of 12 participants across the groups did not complete the study due to inability to fall asleep or equipment error (PMR: five; control: eight). For some participants, due to poor EEG channels, we were unable to evaluate power spectra, thus degrees of freedom denote the total number of participants included.

Participants were administered five questionnaires, the Horne‐Östberg (HO), Epworth Sleepiness Scale (ESS), Beck‐Depression Inventory (BDI‐II), Karolinska Sleepiness Scale (KSS), and STAI (Beck et al., 1996; Horne & Östberg, 1976; Johns, 1991; Shahid et al., 2011; Spielberger, 2010). One participant did not complete any of the questionnaires, four other participants did not complete the STAI (three from PMR and two from control). Two participants did not complete the KSS (one from PMR and one from control). Two participants from each group did not complete the BDI.

### 2.2 Experimental design

**FIGURE 1:**
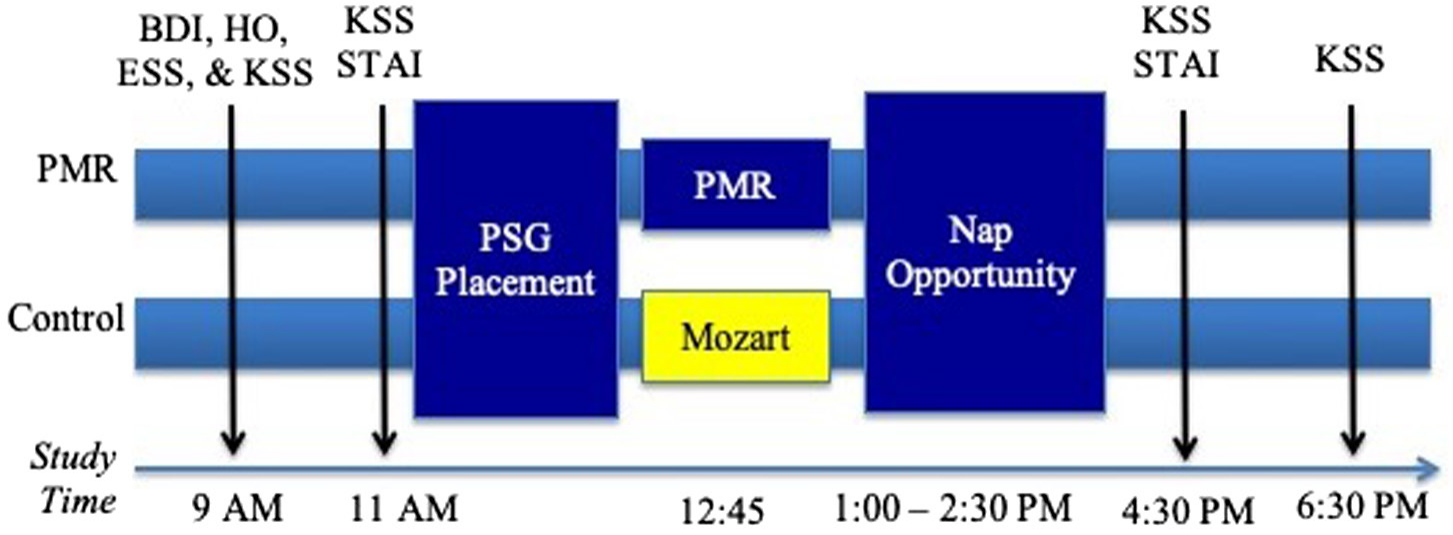
Study timeline. Participants arrived at the laboratory in the morning, completed questionnaires a 2 and 4 h before and after the nap. Participants were randomly assigned to engage in the progressive muscle relaxation (PMR) training or listen to a medley of Mozart music

We designed our study to understand the effects of PMR on daytime sleep architecture and sleeping brain rhythms (Figure 1 for experimental design). All participants arrived at the laboratory at 8:30 a.m. to be consented. Approximately 4 h before the nap, participants completed the KSS, HO, BDI, and ESS questionnaires. Just before polysomnography (PSG) administration, ~2 h before the nap, participants were administered the KSS again and the STAI. After PSG administration, participants randomly assigned to the PMR intervention group laid in bed and were instructed to listen to and follow along with a pre‐recorded 10‐min training that led them through actively tensing and releasing the muscles across their body. Those in the active control laid in the bed awake and quietly listened to a medley tape of classical Mozart music, chosen to be relaxing, for an equivalent 10‐min period. Participants napped at ~1:00 p.m. in the afternoon. After the nap, participants completed the KSS and STAI 2 h after the nap and again 4 h after the nap.

### 2.3 Questionnaires

We administered five questionnaires to assess circadian preference, general sleepiness, fatigue, and anxiety. To assess circadian preference, we administered the HO questionnaire, which includes 19 questions, of which provides scores ranging from 16 to 86 and indicate whether the individual falls in the following categories: definite evening, moderate evening, intermediate, moderate morning, and definite morning (Horne & Östberg, 1976). To assess general sleepiness, we administered the ESS, with participants rating a series of activities on a 0–3 scale indicating how likely they are to fall asleep (Johns, 1991). Higher total scores, ranging from 0 to 24, are indicative of greater sleepiness. To assess depression, we administered the BDI‐II, which is a 21‐item questionnaire asking participants to self‐report depressive symptoms, with higher scores suggesting the presence of depression. We repeatedly measured the KSS, a measure of subjective sleepiness on a scale from 1 to 9, with 1 being alert and 9 being extremely sleepy, four times to all participants (Shahid et al., 2011). This provided us with the opportunity to assess changes in subjective sleepiness across the day. We also administered the STAI‐State subscale twice to identify the degree of anxiety experienced by participants before and after a nap (Spielberger, 2010). These questions are administered on a 4‐point scale with total higher scores implicating greater amounts of anxiety in the moment.

### 2.4 Polysomnography

We administered a four single‐electrodes polysomnography montage to our participants including EEG, electro‐oculography (EOG), and electromyography (EMG) according to the international 10–20 System (Jasper, 1958). We cross referenced the scalp EEG and EOG electrodes to the opposite mastoid (A1 and A2). PSG was recorded using the Astro‐Med Grass Heritage Model 15 amplifiers set at a 256‐Hz sample rate with Grass Gamma software. We applied low‐ (0.3 Hz) and high‐pass filters (35 Hz) to all EEG and EOG channels. Two blind‐to‐group raters scored each participants’ raw sleep EEG data according to American Academy of Sleep Medicine (AASM) guidelines in 30‐s epochs using four, single scalp electrodes (C3, C4, O1, O2), EMG, and EOG. As we did not have frontal electrodes, we determined the presence of slow waves using the central electrodes. All artefacts and arousals were removed via visual inspection. For each participant, we calculated the TST, SOL (minutes between lights out and first epoch of sleep), WASO, sleep efficiency, and the minutes spent in each sleep stage (wake, N1, N2, N3 [referred to as SWS], and REM).

### 2.5 Power spectrum

To examine the impact of relaxation interventions compared to control group on the power spectrum of the nap, we analysed each participant’s power spectra at C3 and C4, including slow‐wave activity ([SWA], 0.3–1 Hz), delta (1–4 Hz), theta (4–7 Hz), alpha (8–12 Hz), or sigma (12–15 Hz). For each participant, we first removed EEG epochs contaminated by muscle movement and/or other artefacts by applying a simple out‐of‐bounds test (±200 µV threshold) on the high‐pass filtered (0.5 Hz) version of the EEG signals. We then computed the EEG power spectra for the central electrodes (C3 and C4) using Matlab for each participant using the Welch method (4‐s Hanning windows with 50% overlap) on the artefact‐free 30‐s epochs. Lastly, for each sleep stage (N2 and SWS), we averaged the power spectra across the central electrodes (C3 and C4) SWA, delta, theta, alpha, and sigma.

### 2.6 Statistical analyses

To examine the impact of relaxation interventions on general sleep variables and daytime napping architecture, we ran one‐way ANOVAs. One variable, time spent in REM sleep, did not satisfy the equality of variance assumption, thus we analysed this difference using a Welch ANOVA. We also compared the time it took to enter each stage within the nap (latency to each stage) and the time it took to enter into consolidated sleep, defined as the latency time to a full 10 min of sleep. For N1, N2, and SWS, we also ran one‐way ANOVAs for each power spectra range, including SWA, delta, theta, alpha, and sigma. To examine EEG laterality, for each sleep stage we created an index score of right hemisphere activity compared to left hemisphere activity (C3 – C4) for SWA and delta activity, where scores >0 indicate left‐lateralised EEG and <0 indicate right‐lateralised EEG. We ran one‐way ANOVAs separately for each sleep stage and frequency bands to examine group laterality differences. To examine changes in both KSS and STAI scores between morning and evening, we ran repeated measure ANOVAs of Time (for KSS: a.m.1, a.m.2, p.m.1, and p.m.2; for STAI: a.m. and p.m.) by group (PMR, control). Lastly, to assess the relationship between sleep architecture and subjective ratings of sleepiness and state‐anxiety for each group, we ran correlations between KSS and STAI scores and sleep variables (time in stages and power). For all variables, outliers were considered 3 SDs above the mean.

## 3 RESULTS

### 3.1 Sleep

General sleep data for all two groups is presented in Table 1. With respect to general napping features, participants had similar TST (F[1,36] = 0.507, *p* = 0.481; mean [SE] PMR group 66.44 [4.81] and control 71.41 [5.06] min), SOL (F[1,36] = 0.113, *p* = 0.739; mean [SE] PMR group 6.81 [1.04] and control 7.33 [1.13] min), WASO (F[1,36] = 0.027, *p* = 0.870; mean [SE] PMR group 12.65 [3.49] and control 13.55 [4.19] min), and sleep efficiency (F[1,36] = 0.003, *p *= 0.960; mean [SE] PMR group 77.76% [3.57%] and control 78.05% [4.51%]).

**TABLE 1:** Reflects general sleep architecture values in minutes, parentheses denote standard error (SE) for total sleep time (TST), sleep onset latency (SOL), wake after sleep onset (WASO), sleep efficiency, and time spent in each sleep stage

	Progressive muscle relaxation (PMR)	Control
	Mean (SE)	Mean (SE)
TST	66.44 (4.81)	71.41 (5.06)
SOL	6.81 (1.04)	7.33 (1.13)
WASO	12.65 (3.49)	13.55 (4.19)
Sleep efficiency	77.76 (3.57)	78.05 (4.51)
N1	8.15 (1.37)	8.36 (1.4)
N2	35.68 (3.35)	41.72 (3.81)
SWS	18.57 (3.26)*	9.22 (2.59)
REM	4.026 (1.64)*	12.05 (2.96)

SWS, slow‐wave sleep; REM, rapid eye movement.

Asterisks denote that the progressive muscle relaxation intervention group spent significantly greater time in SWS and less in REM compared to the active control group (*p* < 0.05).

**FIGURE 2:**
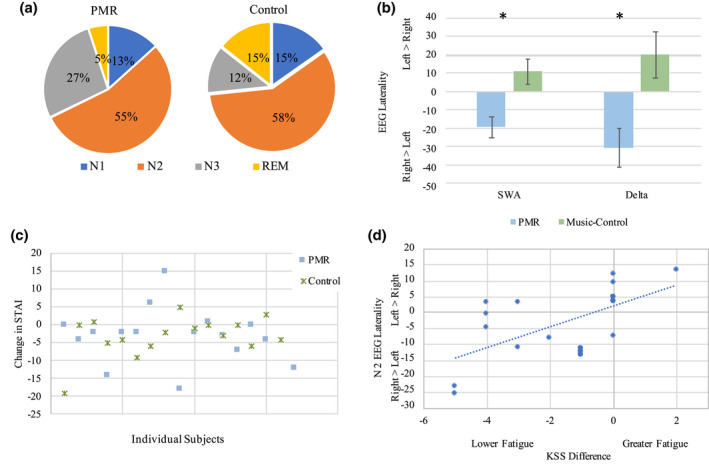
(a) The average amount of time spent in each stage as a percentage of the total nap. Participants spent 125% more time in slow‐wave sleep (SWS) in the progressive muscle relaxation (PMR) than control group. (b) Participants in the PMR group had greater right‐lateralised slow‐frequency activity compared to left‐lateralised in the control group. (c) Participants’ difference between before and after nap anxiety is plotted. Each marker represents an individual participant. We found an overall decrease after the nap, but no differences between groups. (d) Participants whose showed lower fatigue after the nap also had greater right‐lateralised electroencephalography (EEG) activity. KSS, Karolinska Sleepiness Scale; REM, rapid eye movement; SWA, slow‐wave activity; STAI, State‐Trait Anxiety Inventory

Time spent in stages can be seen in Table 1. Participants in the PMR intervention spent significantly more time in SWS (F[1,36] = 4.916, *p* = 0.033; mean [SE] PMR group 18.57 [3.26] and control 9.22 [2.59] min) but significantly less time in REM (F[1,36] = 5.772 *p* = 0.022; mean [SE] PMR group 4.026 [1.64] and control 12.05 [2.96] min) than the active control group (Figure 2A). This was a 10 min difference, equivalent to 125% more total nap time spent in SWS in the PMR than the control group (Figure 2). There were no significant time differences between groups for N1 (F[1,36] = 0.010, *p* = 0.919; mean [SE] PMR group 8.15 [1.37] and control 8.36 [1.4] min) or N2 (F[1,36] = 1.422, *p* = 0.241; mean [SE] PMR group 35.68 [3.35] and control 41.72 [3.81] min). There were also no significant differences across the two groups between the first epoch of sleep and entering N1, N2, or SWS (all *p* > 0.739), suggesting that despite amount differences, the general sleep architecture patterns remains similar up to SWS.

### 3.2 Power analyses

We evaluated N2 and SWS separately for power spectra differences at central electrodes (average C3 and C4) in SWA, delta, theta, alpha and sigma (see Table 2 for mean and SE values). We found no significant differences in the total amount of power for either sleep stage in SWA (N2: F[1,32] = 0.098, *p* = 0.756, mean [SE] PMR group 57.29 [5.96] and control 53.86 [9.64]; SWS: F[1,26] = .010, *p* = 0.920, mean [SE] PMR group 175.96 [20.42] and control 172. 57 [26.95]), delta (N2: F[1,32] = 0.00, *p* = 0.984, mean [SE] PMR group 128.69 [11.51] and control 128.21 [22.33]; SWS: F[1,26] = 0.052, *p* = 0.920, mean [SE] PMR group 341.2 [32.3] and control 354.41 [52.24]), theta (N2: F[1,32] = 0.004, *p* = 0.952, mean [SE] PMR group 9.74 [1.1] and control 9.83 [0.922]; SWS: F[1,26] = 2.126, *p* = 0.157, mean [SE] PMR group 10.04 [1.29] and control 12.57 [0.89]); alpha (N2: F[1,32] = 0.013, *p* = 0.721, mean [SE] PMR group 21.06 [2.21] and control 22.47 [3.37]; SWS: F[1,26] = 1.341, *p *= 0.258, mean [SE] PMR group 27.03 [2.68] and control 32.64 [4.35]) or sigma (N2: F[1,32] = 0.00, *p* = 0.992, mean [SE] PMR group 4.8 [0.55] and control 4.79 [0.461]; SWS: F[1,26] = 0.017, *p* = 0.899, mean [SE] PMR group 4.39 [0.609] and control 4.49 [0.299]; Table 3).

**TABLE 2:** Spectral power values for each stage and group are presented. No significant differences were found across groups

		Progressive muscle relaxation (PMR)	Control
		Mean (SE)	Mean (SE)
N2	SWA	57.29 (5.96)	53.86 (9.64)
	Delta	128.69 (11.51)	128.21 (22.33)
	Theta	9.74 (1.1)	9.83 (0.922)
	Alpha	21.06 (2.21)	22.47 (3.37)
	Sigma	4.8 (0.55)	4.79 (0.461)
SWS	SWA	175.96 (20.42)	172.57 (26.95)
	Delta	341.2 (32.3)	354.41 (52.24)
	Theta	10.04 (1.29)	12.57 (0.89)
	Alpha	27.03 (2.68)	32.64 (4.35)
	Sigma	4.39 (0.609)	4.49 (0.299)

SWA, slow‐wave activity; SWS, slow‐wave sleep.

**TABLE 3:** Electroencephalography (EEG) laterality for low‐frequency power for each stage. For each sleep stage, we created an index score of right hemisphere activity compared to left hemisphere activity (C3 – C4) for slow‐wave activity (SWA) and delta activity, where scores >0 indicate left‐lateralised EEG and <0 indicate right‐lateralised EEG

		Progressive muscle relaxation (PMR)	Control
		Mean (SE)	Mean (SE)
N2	SWA	−4.15 (2.61)	−1.41 (2.31)
	Delta	−6.75 (5.31)	−2.61 (4.18)
SWS	SWA	−19.42 (5.72)*	10.65 (6.81)
	Delta	−30.69 (10.51)*	19.73 (12.3)

In slow‐wave sleep (SWS), we found significantly greater SWA and delta power in the PMR condition. Means and standard errors are reported.

Asterisks denote a significant difference between PMR and control (*p* < 0.05).

### 3.3 SWA and delta laterality

We found significant differences in the lateralisation of central low spectral frequency power between groups in SWS but not N2. In SWS, in both SWA and delta bands, PMR participants had greater right‐lateralised activity than the control group in the SWA band (F[1,26] = 11.365, *p* = 0.002; mean [SE] PMR group −19.42 [5.72] and control 10.65 [6.81]) and delta band (F[1,26] = 9.598, *p* = 0.005; mean [SE] PMR group −30.69 [10.51] and control 19.73 [12.3]; Figure 2B). We did not find laterality differences in N2 between the groups for SWA (F[1,32] = 0.590, *p* = 0.448; mean [SE] PMR group −4.1553 [2.61] and control −1.41 [2.31]) or delta (F[1,32] = 0.357, *p* = 0.554; mean [SE] PMR group −6.775 [5.31] and control –2.61 [4.18]).

### 3.4 Questionnaires

We ran a repeated measures ANOVA on the KSS with Time (a.m.1, a.m.2, p.m.3, p.m.4) by Group (PMR, Control). We found a significant main effect of Time (F[3,33] = 18.19, *p *< 0.001) but no interaction between Time and Group. Post hoc analyses revealed greater sleepiness in the morning than afternoon post‐napping (comparing 2 h before the nap to 2 h after the nap, *p* < 0.001). We also ran a repeated measures ANOVA on the STAI for Time (a.m., p.m.) by Group (PMR, Control). We found a significant main effect of Time on the STAI (F[1,15] = 8.812, *p *= 0.01), but no differences between groups (see Table 4 for means and SE). Post hoc analyses revealed that after the nap, participants’ STAI scores decreased (see Figure 2C for plot of difference scores). Lastly, we ran one‐way ANOVAs on participants’ HO, ESS, and BDI scores. We found no significant differences between groups for any of the three questionnaires (HO: F[1,35] = 0.536, *p* = 0.469; ESS: F[1,35] = 584, *p* = 0.45; BDI: F[1,32] = 0.025, *p* = 0.875).

**TABLE 4:** Reflects change in subjective rating of sleepiness (KSS) and anxiety (STAI) across the day, and general circadian alignment, sleepiness upon arrival, and depression, across participants. Mean and standard error (SE) values are reported

	Progressive muscle relaxation (PMR)	Control
	Mean (SE)	Mean (SE)
KSS a.m.1	3.83 (0.41)	3.33 (0.37)
KSS2 a.m.2	5.56 (0.36)	4.76 (0.38)
KSS3 p.m.1	3.33 (0.31)	2.72 (0.32)
KSS4 p.m.2	3.72 (0.39)	2.88 (0.37)
STAI a.m.	28.88 (1.64)	31.81 (1.62)
STAI p.m.	25.88 (2)	28.69 (1.42)
HO	46.88 (1.92)	49.02 (2.27)
ESS	7.16 (0.72)	6.5 (0.51)
BDI	3.76 (1.12)	4 (1.03)

BDI, Beck‐Depression Inventory; ESS, Epworth Sleepiness Scale; HO, Horne‐Östberg questionnaire; KSS, Karolinska Sleepiness Scale; STAI, State‐Trait Anxiety Inventory.

### 3.5 Relationship between subjective ratings and sleep architecture

**FIGURE 3:**
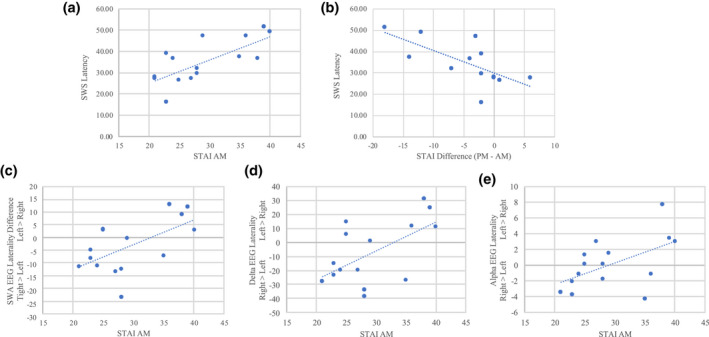
Progressive muscle relaxation (PMR) group correlations. (a) Morning anxiety positively correlated with slow‐wave sleep (SWS) latency. (b) The change in anxiety correlated negatively with SWS latency. Graphs (c–e) Morning anxiety levels correlated with increased left‐lateralised electroencephalography (EEG) activity in slow‐wave activity (SWA), delta, and alpha bands. STAI, State‐Trait Anxiety Inventory

We evaluated the relationship between the nap sleep architecture and subjective ratings of sleepiness (KSS) or state‐anxiety (STAI) both before and after the nap. In the PMR group, we found that SWS latency positively correlated with the morning STAI score (*r* = 0.725, *p* = 0.002) and negatively correlated with the anxiety change after nap (*r* = −0.68, *p* = 0.006) (Figure 3). WASO also negatively correlated with the percentage of nap time spent in SWS (*r* = −0.62, *p* = 0.004), while sleep efficiency positively correlated with the percentage of SWS sleep in the nap (*r* = 0.549, *p* = 0.014). We then evaluated the relationship between anxiety and fatigue with EEG power, specifically the lateralisation of low‐frequency bands and alpha for N2 and SWS. In N2, greater morning anxiety was associated with increased left‐lateralised SWA (*r* = 0.595, *p* = 0.019), delta activity (*r* = 0.589, *p* = 0.02), and alpha activity (*r* = 0.599, *p* = 0.023). Lastly, greater left‐lateralised SWA activity in N2 correlated with the change in fatigue (difference in KSS score following versus preceding sleep) over the nap (*r* = 0.601, *p* = 0.011), such that participants who showed lower fatigue after the nap also had greater right‐lateralised EEG activity (Figure 2D).

In the control group, we found that morning and afternoon anxiety negatively correlated with the percentage of time spent in N2 (a.m.: *r* = −0.87, *p* < 0.001; p.m.: *r* = −0.52, *p* = 0.038), while percentage of time spent in SWS during the nap was positively correlated with the change in anxiety from pre‐ to post‐nap (*r* = 0.555, *p* = 0.03). We found no relationships between power spectra and anxiety or fatigue.

## 4 DISCUSSION

In the present study, our goal was to investigate if PMR, an accessible, physiologically‐based relaxation intervention, before a daytime nap would result in the same restorative sleep benefits during a nap compared to listening to classical music. Compared to active control, PMR resulted in increased time spent in SWS, suggesting similar benefits from PMR for napping as for night‐time sleep. This 10 min difference was equivalent to 125% more total napping time spent in SWS, which comprised ~27% of the total nap in the PMR group compared to only 12% of the nap time in the control group. This SWS enhancement was concomitantly related to a reduction in the time spent in REM, with PMR participants spending 5% compared to 15% of the total nap time in REM in the control group. Sleep stage latency for Stages 1, 2, and SWS was similar for both groups, suggesting that the initial overall sleep architecture patterns were similar, but the length of SWS is what differed. Morning anxiety state also affected subsequent sleep architecture, correlating both with subsequent SWS latency and EEG lateralisation. As such, our results suggest a trade‐off between SWS and REM sleep driven by the pre‐nap relaxation intervention with potential implications for clinically anxious populations.

Although we found no total power spectra differences between groups, our intervention affected the laterality of low‐frequency power. In SWS, PMR participants had greater right‐lateralised SWA and delta activity, whereas control participants showed greater left‐lateralised activity. This suggests the possibility that PMR induced a degree of restoration that reduced the requirements during sleep and, consequently, less left‐lateralised activity during SWS. Prior studies have revealed similar results. A study by Pifarré et al. with oncological patients using ^18^F‐fluorodeoxyglucose‐positron emission tomography showed that PMR resulted in widespread cortical glucose consumption reduction as compared to a resting control (Pifarré et al., 2015). Interestingly, this reduced glucose consumption mirrored that of diazepam, suggesting that psychological relaxation methods could result in similar anxiolytic‐induced relaxed brain states. In conjunction, a functional magnetic resonance imaging study showed that repeatedly engaging in PMR, compared to an active physiological tensing control, resulted in reduced limbic and cerebral cortical activity (Kobayashi & Koitabashi, 2016). Together, these studies suggest that PMR results in mirroring physiological and neurological reduced stress states. Thus, it is a possibility that our PMR intervention induced alleviated exhausted neurological circuits resulting in a more well‐rested brain profile during sleep.

The relationship between morning state anxiety and sleep is more perplexing. Across the study, all participants’ anxiety benefited from the nap. However, specific to the PMR group, SWS latency correlated with both pre‐ and post‐sleep anxiety ratings. As such, participants who took longer to enter into SWS reported higher baseline anxiety and greater post‐nap reductions in anxiety. Previous literature has shown that greater daily stress increases SWS latency; however, with PMR this is also related to a lowering of anxiety (Åkerstedt et al., 2007). In conjunction, greater morning anxiety in the PMR group was predictive of greater left‐lateralised EEG activity during the nap. Greater waking right‐lateralised alpha activity has been linked to depression and anxiety (Coan & Allen, 2004). The relationship between anxiety and SWA and delta EEG lateralisation during sleep is less clear and should be the focus of future studies. It is possible that our use of healthy, non‐disordered participants and between‐subject design impacted our ability to tease apart this relationship further. As anxiety symptoms are known to impact sleep architecture in disordered populations, specifically increasing SOL and decreasing the total amount of NREM and REM (Horváth et al., 2016), it would be interested to evaluate if PMR before a nap mitigates this NREM deficit.

There are some limitations that should be considered when evaluating our findings. We employed a between‐subject, single‐intervention design using a non‐clinical cohort. As a follow‐up, it will be important to investigate PMR before a nap using a within‐subject design. This will also give the opportunity to investigate how participants rate their subjective sleep quality after the PMR and control naps, with the goal of identifying potential mechanisms of sleep misperception that can be translated to sleep disorder research (Edinger & Fins, 1995). Although we found that morning anxiety was correlated with greater left‐lateralised delta and alpha activity, we did not find differences between pre‐ and post‐nap anxiety scores. Thus, it will also be important to evaluate if these differences remain consistent using a within‐subject design and in clinical populations. Our present study was also not designed to evaluate the effects of music on sleep architecture, and we did not ask participants about their feelings of relaxation after the intervention nor investigated how aspects of the music (i.e., beats/min or rhythm) might affect the pre‐sleep waking state (Dickson & Schubert, 2019). Prior research has found that music can increase REM‐rich sleep overnight in a clinical population (Chang et al., 2012). Future investigations could evaluate if music can similarly enhance REM during a nap and should include subjective measures of music enjoyment and relaxation. Lastly, given that PMR is also frequently trained over multiple sessions in clinical populations, it will be interesting to evaluate the effects on sleep over longer periods of time (Conrad & Roth, 2007). We did not assess prior music or PMR training, thus are unable to identify if prior training impacted our results. The feasibility and accessibility of PMR before a nap make this an interesting research avenue to pursue with strong translational application.

## 5 CONCLUSION

Our study demonstrated that a PMR relaxation‐based intervention before daytime napping shifts sleep architecture towards a SWS‐rich nap. Our observation of intervention associated right‐lateralised predominant activity suggests the possibility that even a short PMR session could support restorative function such that the nap demonstrates a more well‐rested brain profile. This ability to induce SWS‐rich sleep with a brief, accessible relaxation intervention creates strong opportunity for translational application. Given the frequency of chronic sleep restriction in the general population and long‐term physical and cognitive consequences, enhancing the quality or type of sleep in a nap could be beneficial.

## CONFLICT OF INTEREST

The authors have declared no conflict of interest.

## AUTHOR CONTRIBUTIONS

Katharine C. Simon: analysed the data and wrote the manuscript. Elizabeth A. McDevitt and Rocco Ragano: collected the data, assisted with study design. Sara C. Mednick: edited the manuscript and designed the study.

## Data Availability

Data available on request from the authors.
